# Structural determinants of Ca_V_1.3 L-type calcium channel gating

**DOI:** 10.1186/1471-2210-11-S2-A11

**Published:** 2011-09-05

**Authors:** Andreas Lieb, Anja Scharinger, Florian Hechenblaickner, Mathias Gebhart, Alexandra Koschak, Martina J Sinnegger-Brauns, Jörg Striessnig

**Affiliations:** 1 Department of Pharmacology and Toxicology, Institute of Pharmacy, and Center for Molecular Biosciences Innsbruck (CMBI), University of Innsbruck, 6020 Innsbruck, Austria

## Background

Ca_V_1.3 channels, which belong to the family of voltage-gated L-type calcium channels (LTCCs), are involved in important physiological (e.g. hearing, hormone release and cardiac and neuronal pace making) and pathophysiological functions (e.g. Parkinson’s disease). We have recently discovered that an intramolecular protein interaction within the C-terminus of Ca_V_1.3 α1 subunits fine-tunes Ca_V_1.3 channel function. This C-terminal modulatory mechanism (CTM) is present in the long (Ca_V_1.3_l_) but is absent in the short (Ca_V_1.3_42A_) splice variant. Its absence induces activation at a more negative voltage range and increases Ca^2+^-dependent inactivation (CDI). Interestingly a functional CTM is present in the human [1] and rat Ca_V_1.3 α1 subunit isolated from pancreatic islets (D38101, rCa_V_1.3_pan_) but not in a rat Ca_V_1.3 α1 subunit cDNA clone isolated from superior cervical ganglion (scg) (AF370010; rCa_V_1.3_scg_). This causes substantial differences in the voltage- and Ca^2+^-dependent gating of scg and pan.

## Methods

We systematically compared scg and pan Ca_V_1.3 α1 subunits by expression in tsA201 cells and analysis of their functional properties using the whole-cell patch-clamp technique, to determine the structural basis for this difference.

## Results

rCa_V_1.3_scg_ differs from rCa_V_1.3_pan_ at three amino acid positions (S244G, V1104A, A2073V) and one alternatively spliced locus (absence of exon 31). Alternative splicing did not explain the functional differences between the two rCa_V_1.3 α1 subunits. The amino acid difference A2073V is located within the recently identified distal part (DCRD) of a C-terminal modulatory domain. Mutation of A2073 in rCa_V_1.3_scg_ to the corresponding valine (A2073V) in rCa_V_1.3_pan_ fully restores the slower CDI of rCa_V_1.3_pan_. In contrast, A2073V only weakly affected the activation voltage range (rescue of only 5.3 mV of the 17.2 mV difference in the half-maximal voltage activation range (V_h_)). Additional mutation of S244 to G in the rCa_V_1.3_scg_ S4-S5 linker of domain I caused a further shift to a more positive voltage close to the V_h_ of rCa_V_1.3_pan_.

## Conclusions

Our data identify residues at proposed interfaces between voltage sensors and the intracellular channel gate controlling the voltage-dependence of Ca_V_1.3 activation. We also show that the DCRD domain can moderate CDI independently of its effect on V_h_, suggesting that these processes occur through different DCRD-dependent mechanisms.
